# Three-year clinical outcome of XEN45 Gel Stent implantation versus trabeculectomy in patients with open angle glaucoma

**DOI:** 10.1038/s41433-024-03042-z

**Published:** 2024-03-28

**Authors:** Teresa Rauchegger, Sarah-Maria Krause, Yvonne Nowosielski, Anna Lena Huber, Peter Willeit, Eduard Schmid, Barbara Teuchner

**Affiliations:** 1 Department of Ophthalmology and Optometry, Medical University of Innsbruck, Innsbruck, Austria; 2 Institute of Health Economics, Medical University of Innsbruck, Innsbruck, Austria; 3 Department of Public Health and Primary Care, University of Cambridge, Cambridge, UK

**Keywords:** Glaucoma, Conjunctival diseases

## Abstract

**Objective:**

To reliably compare the three-year clinical outcome and safety of XEN45 Gel Stent implantation (XEN) vs. trabeculectomy (TRAB) in patients with glaucoma.

**Subject/methods:**

We conducted a retrospective cohort study with patients with primary open angle or pseudoexfoliation glaucoma with uncontrolled intraocular pressure (IOP) undergoing XEN or TRAB at the Innsbruck University Clinic of Ophthalmology and Optometry, Austria and analysed changes in IOP, numbers of IOP-lowering medications, and complete surgical success (i.e., IOP ≤ 18 mmHg, ≥20% IOP reduction and not requiring IOP-lowering medication) up to 36 months postoperatively.

**Results:**

Between 2013 and 2019, we performed XEN Gel Stent implantation in 58 eyes and trabeculectomy in 84 eyes. From baseline to 36 months, mean IOP decreased from 23.4 to 13.8 mmHg (mean reduction 35%, 95% confidence interval 23–48%, *p* < 0.001) in the XEN group and from 25.1 to 11.2 mmHg (mean reduction 50%, 41–60%, *p* < 0.001) in the TRAB group. TRAB provided higher IOP reduction than XEN Gel Stent implantation at 12, 24, and 36 months (all *p* < 0.05). In XEN versus TRAB, IOP-lowering medication was required by 98.3% vs. 97.6% before surgery (*p* = 0.781), differed significantly at month 12 (43.2% vs. 2.0%, *p* < 0.001)but not at month 24 or 36. Complete surgical success was achieved in 40.0% vs. 62.8% at month 24 (adjusted odds ratio 2.70; 1.04–7.00, *p* = 0.040) and 27.3% vs. 56.8% at month 36 (4.36; 1.25–15.18, *p* = 0.021).

**Conclusion:**

Compared to XEN, TRAB was associated with lower intraocular pressure, less IOP-lowering medication, and higher probability of achieving complete surgical success over a 36-month follow-up period.

## Introduction

The ultimate goal in glaucoma treatment is to prevent a vision-related decrease in quality of life [1]. The main approach for preventing progression is based on lowering intraocular pressure (IOP) to an individual target level. Topical therapy is usually the first line therapy, but, in case medical therapy does not provide adequate IOP reduction, glaucoma surgery becomes necessary [2].

With the introduction of minimally invasive glaucoma surgery (MIGS) and devices of enhanced subconjunctival glaucoma surgery, treatment options broadened, but trabeculectomy is still considered the gold standard [3, 4]. Among the different devices available to date, the XEN Gel Stent (XEN® 45, Allergan, an Abbvie company, Irvine, CA, USA) allows subconjunctival filtration similar to trabeculectomy. The stent is a hydrophilic tube made of porcine gelatine cross-linked with glutaraldehyde, with an inner lumen of 45 μm and a length of 6 mm. It is placed through a clear cornea incision in the subconjunctival space at the superonasal quadrant and lowers IOP by creating an outflow pathway from the anterior chamber to the subconjunctival or subtenon space that bypasses the trabecular meshwork [5, 6].

Different studies have been published demonstrating the short-term benefits and safety of XEN Gel Stent application in patients with open angle glaucoma [7–9]. However, results from prospective randomised controlled trials comparing trabeculectomy and XEN Gel Stent implantation are missing [10], and there are only a few published retrospective studies with follow-up data longer than one year [11–13]. Therefore, in our study, we aimed to compare in detail the long-term clinical outcome and safety of XEN Gel Stent implantation vs. trabeculectomy for primary open angle glaucoma and pseudoexfoliation glaucoma.

## Subjects and methods

### Study design

This is a retrospective cohort study of 142 eyes in 124 consecutive patients presenting between 2013 and 2019 at the University Clinic of Ophthalmology and Optometry of the Medical University of Innsbruck, Austria, with medically uncontrollable IOP due to primary open angle glaucoma or pseudoexfoliation glaucoma. XEN Gel Stent implantation (XEN) or trabeculectomy (TRAB) was performed by two equally highly experienced glaucoma surgeons (B.T., E.S.). The study followed the tenets of the Declaration of Helsinki, and its protocol was approved by the local ethics committee under the reference number 1333/2020.

### Inclusion and exclusion criteria

Patients aged 45 or older with either primary open-angle or pseudoexfoliation glaucoma, which uncontrolled intraocular pressure on maximally tolerated medical therapy, were included. Patients who underwent previous ocular surgeries were excluded, except from previous glaucoma surgeries and phacoemulsification with IOL implantation performed more than 6 months before the filtration surgery.

### Baseline and follow-up data collected in our study

Preoperative baseline characteristics were collected from electronic medical files, and included the ocular characteristics type of glaucoma, prior surgical procedures, IOP measured with Goldmann applanation tonometry, best corrected visual acuity (BCVA) in Snellen and visual field (VF) mean deviation in Octopus 900 perimeter (Haag-Streit Diagnostics, Köniz, Switzerland), as well as demographic features of the patients. Follow-up data was obtained through chart review. The data collected postoperatively included IOP readings, number of glaucoma medication, BCVA, number of complications, number of needling procedure and, if applicable, type of secondary IOP-lowering procedure. Data were collected up to 3 years postoperatively, ie after 1 day, 1 week, 1 month, 3 months, 6 months, 12 months, 18 months, 24 months, 30 months, and 36 months.

### XEN gel stent implantation

Surgery was performed under local anaesthesia. An eyelid retractor was inserted and the target sector was marked in the nasal upper quadrant of the conjunctiva 3–4 mm from the limbus. Then, an antimetabolite (0.1 ml of diluted Mitomycin C (0.028 mg/ml)) was injected subconjunctivally adjacent to the target sector. If a cataract surgery was planned, a routine phacoemulsification with intraocular lens implantation was performed following the antimetabolite injection and prior to XEN implantation. Two corneal paracenteses were created. The anterior chamber was maintained with the use of a viscoelastic substance (Healon Pro; Johnson & Johnson Vision, Santa Ana, CA, USA) before the 27-gauge preloaded injector (XEN® 45, Allergan, an Abbvie company, Irvine, CA, USA) was inserted through the paracentesis at the inferotemporal quadrant. The eye was stabilised with a Vera Hook through the second paracentesis. The stent was implanted at the superonasal part of the anterior chamber angle aiming for the 3 mm mark using the preloaded injector. Once the bevel of the needle was visible under the conjunctiva/subtenon space the injector was rotated and the XEN Gel Stent was injected in the subconjunctival space. The position of the inner part of XEN Gel Stent was ensured by gonioscopy. The mobility of the subconjunctival part of the stent was checked by moving the end of the implant slightly from side to side under the conjunctiva. The viscoelastic was washed out by irrigation aspiration with balanced salt solution (BSS) and the implant function and the formation of the bleb was checked. The corneal incisions were hydrated with BSS. On the day of surgery, all local and systemic antiglaucoma medications were stopped.

### Trabeculectomy

Trabeculectomy was always performed under retrobulbar anaesthesia. A corneal support suture (7-0 ProleneTM, Ethicon Inc., New Jersey, USA) was applied to rotate the eye downwards. A limbus based conjunctival pocket was fashioned and the subtenon space was prepared. Thin cellulose sponges (LASIK shields, Beaver-Visitec International, West Africa) soaked in 0.028 mg/ml mitomycin C solution (MMC) were placed under the conjunctiva and tenon for 3 min. Light wet field cautery was applied to the areas of bleeding and the flap area. The anterior chamber was opened with a paracentesis and filled with viscoelastic (DisCoVisc®, Alcon GmBH, Germany). A scleral flap (approximately 3 x 3 mm) was prepared and a trabeculectomy was created with a Grehn glaucoma punch (Geuder AG, Germany). A peripheral iridectomy was done and the viscoelastic was rinsed out. The sclera flap was sutured with two to three 10/0 nylon single sutures (MonosofTM, Covidien plc, Dublin, Ireland) to provide sufficient fistulation. The conjunctiva and tenon were closed with a meander-like running suture (“Mainz suture”) with 10/0 nylon at the limbus.

### Postoperative management

On the day of surgery all local and systemic anti-glaucomatous medications were discontinued. Postoperatively, anti-inflammatory eye drops (prednisolone acetate 1% or equivalent) were applied initially every hour and then slowly reduced over several months depending on the appearance of the bleb. Antibiotics (ofloxacin or equivalent) were administered four times daily for one week.

Postoperative needling was performed occasionally as needed, under local topical anaesthesia by sweeping a 27-gauge needle in the subconjunctival space in order to dissect subconjunctival and subtenon scarring. At the end of the procedure, a volume of 0.05 ml of diluted MMC solution (0.1 mg/ml) was injected in the newly formed subtenon space.

Bleb revision was performed under retrobulbar anaesthesia. The conjunctiva and tenon were opened at the limbus and the bleb scarring was carefully dissolved. In case of trabeculectomy, the fistulation was checked and if necessary, scleral flap and trabeculectomy was re-opened and new sutures (10/0 nylon) were placed. In case of XEN Gel stent, if necessary, the stent was flushed to restore fistulation, Cellulose sponges soaked in 0.028 mg/ml MMC were placed under the conjunctiva and tenon for 3 min. Conjunctiva and tenon were closed with a meander-like running suture (“Mainz suture”) with 10/0 nylon at the limbus.

Laser suture lysis of the scleral sutures was performed with an Argon Laser (Pascal Streamline©, Geodis Austria GmbH, Vienna, Austria) if necessary. Tetracaine eye drops were applied, and the sutures were located using a Hoskins Lens. One to two laser spots were applied (500 mW, 100 ms, 100 μm spot diameter) to open the suture.

### Outcome measures

The primary outcome measure was IOP at 36-month follow-up. Secondary outcomes included the number of postoperative IOP-lowering medications, postoperative complications, additional glaucoma interventions and surgical success rates. Surgical success was defined as postoperative IOP ≤ 18 mmHg and ≥20% IOP reduction without IOP-lowering medication (complete success) or with/without IOP-lowering medication (qualified success) without a secondary IOP-lowering procedure. Complete surgical failure was defined as the necessity of repeat filtering surgery or loss of light perception. Neither needlings nor bleb revisions were considered failures.

### Data management and statistical analysis

Study data were collected and managed using REDCap, an electronic data capture tool hosted at the Medical University of Innsbruck [14, 15]. When analysing baseline characteristics, we summarised categorical variables as numbers (%) and continuous variables as means ± standard deviations and tested for differences between treatment groups using two-sample *t*-tests, *χ*²-tests or Fisher’s exact tests, as appropriate. All continuous variables were approximately normally distributed as shown by non-significant *p*-values from Kolmogorov-Smirnov tests. We used linear regression to estimate mean differences and corresponding 95% confidence intervals (CIs) between the two treatment groups during follow-up in terms of achieved IOP and % changes vs. baseline. In addition, we concomitantly estimated differences in IOP achieved by XEN vs. TRAB throughout the entire follow-up period using a linear mixed model for repeated measurements adjusted for age, sex, number of pre-operative IOP-lowering medications, and phacoemulsification, plus a random intercept allowed to vary at the patient and the eye level. We used *χ*²-tests to compare the proportions of patients requiring IOP-lowering medications as well as those requiring postoperative interventions. We estimated odds ratios for surgical success comparing the TRAB with the XEN group using logistic regression and estimated hazard ratios for suffering surgical failure, requiring a needling, or requiring bleb revision using time-to-event data and Cox proportional hazards regression. For patients suffering surgical failure, all subsequent data including IOP measurements and number of medications were censored. Whenever appropriate, we also employed statistical adjustment for baseline age, sex, number of IOP-lowering medications and prior trabeculectomy. Analyses involved Stata 15.1 MP (Stata Corp, Texas, USA), two-sided *P* values and a significance level of *P* ≤ 0.05.

## Results

A total of 142 eyes that underwent surgery were included in the analysis. In specific, we performed XEN Gel Stent implantation on 58 eyes of 51 patients, trabeculectomy on 84 eyes of 73 patients. Mean age was 74.9 ± 9.4 years (Table 1). 86 (61%) eyes were from female patients and 56 (39%) from male patients. There were no significant differences in most baseline characteristics between the XEN and the TRAB group. However, the mean number of IOP-lowering medications was slightly lower in the XEN group than in the TRAB group (3.2 ± 1.0 vs. 2.6 ± 1.2; *p* = 0.006).

**Table 1:** Baseline characteristics of patients included in the study (*n* = 142).

Variable	Mean ± SD or no. (%)			*P* value
	Total (*n* = 142)	XEN (*n* = 58)	TRAB (*n* = 85)	
Age, years	74.9 ± 9.4	75.2 ± 9.7	74.7 ± 9.3	0.75
Sex				0.76
Female	86 (61%)	36 (62%)	50 (60%)	
Male	56 (39%)	22 (38%)	34 (40%)	
Glaucoma type				0.17
Primary open angle	91 (64%)	41 (71%)	50 (60%)	
Pseudoexfoliation	51 (36%)	17 (29%)	34 (40%)	
IOP, mmHg	24.4 ± 8.4	23.4 ± 8.4	25.1 ± 8.4	0.24
Number of IOP-lowering medications	3.0 ± 1.1	2.6 ± 1.2	3.2 ± 1.0	0.006
Glaucoma severity				0.20
Mild (<=6 dB)	16 (12%)	9 (16%)	7 (9%)	
Moderate ( >6 dB, <=12 dB)	24 (18%)	12 (22%)	12 (15%)	
Advanced ( >12 dB)	94 (70%)	34 (62%)	60 (76%)	
Pseudophakia	109 (77%)	48 (83%)	61 (73%)	0.16
Prior glaucoma surgery				
Prior TRAB	21 (15%)	15 (26%)	6 (7%)	0.003
Prior XEN	6 (4%)	0 (0%)	6 (7%)	0.081
Prior TCP	5 (4%)	1 (2%)	4 (5%)	0.65
Prior ALT/SLT	13 (9%)	3 (5%)	10 (12%)	0.24
Prior trabecular aspiration	1 (1%)	0 (0%)	1 (1%)	1.00

*P* values were calculated using two-sample *t*-tests, χ²-tests or Fisher’s exact tests, as appropriate.

*ALT/SLT* argon laser trabeculopasty/selective laser trabeculopasty, *IOP* intraocular pressure, *TCP* transsceral cyclophotocoagulation, *TRAB* trabeculectomy, *XEN* XEN Gel Stent.

### Intraocular pressure

**Fig. 1:**
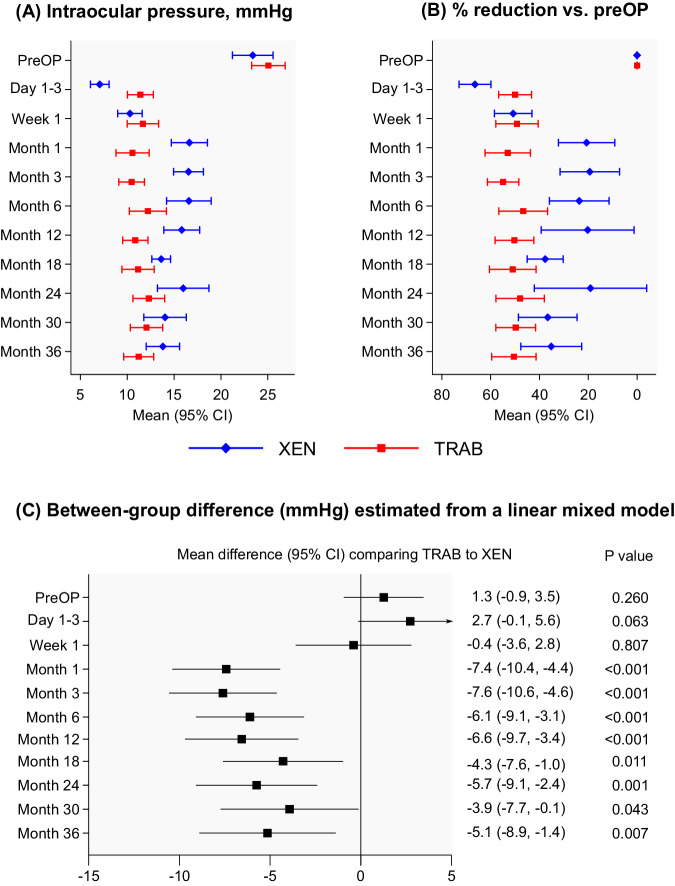
Intraocular pressure (Panel A), within-person percentage reduction compared to the preoperative value (Panel B) in the XEN Gel Stent implantation group and the trabeculectomy group over the 36-month follow-up and adjusted* mean differences in intraocular pressure achieved by XEN vs. TRAB estimated using a linear mixed model that incorporates all repeated measurements during follow-up (Panel C). CI confidence interval, TRAB trabeculectomy, XEN XEN Gel Stent. *The linear mixed model was adjusted for age, sex, number of pre-operative IOP-lowering medications, and phacoemulsification and included a random intercept allowed to vary at the patient and the eye level.

As shown in Fig. 1, IOP was significantly reduced in both treatment groups during the 36-month follow-up period. In the XEN group, mean IOP decreased from 23.4 mmHg at baseline to 16.0 mmHg at 24 months (mean reduction 19%, 95% CI −4 to 42%, *p* = 0.113) and 13.8 mmHg at 36 months (mean reduction 35%, 23–48%, *p* < 0.001). In the TRAB group, mean IOP decreased from 25.1 mmHg at baseline to 12.3 mmHg at 24 months (mean reduction 48%, 38–58%, *p* < 0.001) and 11.2 mmHg at 36 months (mean reduction 50%, 41–60%, *p* < 0.001). Significantly lower IOP values were achieved in the TRAB group compared to the XEN group up to 36 months postoperatively (eg, *p* < 0.001 at month 12; *p* = 0.023 at month 24; *p* = 0.048 at month 36). In a model that incorporates all repeated IOP measurements over time and takes into account for some patients (*n* = 18, 14.5%) both eyes were included, the mean differences in IOP between the TRAB vs. the XEN group remained statistically significant upon adjustment for age, sex, number of medications required at baseline and previous phacoemulsification, i.e. −6.6 mmHg (95% CI −9.7, −3.4; *p* < 0.001) at month 12; −5.7 mmHg (−9.1, −2.4; *p* = 0.001) at month 24; and −5.1 mmHg (−8.9, −1.4; *p* = 0.007) at month 36 (Fig. 1). Notably, there was no evidence that these results differed between patients with primary open angle vs. pseudoexfoliation glaucoma (*P* values for interaction = 0.871). Details on mean IOP measures, percentage reductions, differences between treatments groups, and corresponding 95% CIs are provided in Supplementary Table 1.

### Number requiring intraocular pressure-lowering medication

**Fig. 2:**
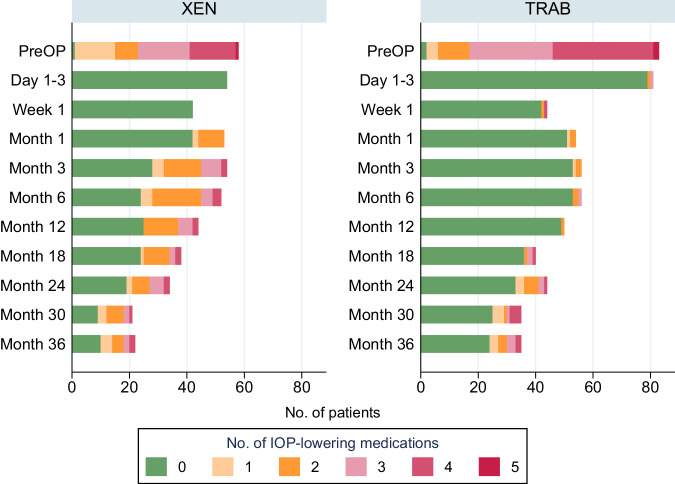
Number of intraocular pressure-lowering medications taken during follow-up. IOP intraocular pressure, TRAB trabeculectomy, XEN XEN Gel Stent.

The percentage of patients in the XEN vs. TRAB group that required IOP-lowering medication was by 98.3% vs. 97.6% before surgery (*p* = 0.781), 43.2% vs. 2.0% at month 12 (*p* < 0.001), 44.12% vs. 25.0% (*p* = 0.076) at 24 month and 54.6% vs. 31.4% at month 36 (*p* = 0.083). A detailed graph on the number of IOP-lowering medication taken over the study visits is provided in Fig. 2. None of the patients received oral acetazolamide after 36-month follow-up.

### Visual acuity (VA) and visual field

At baseline, mean VA was 0.38 (95% CI 0.29, 0.50) in the XEN group and 0.34 (0.28, 0.41) in the TRAB group (*P* = 0.518) (Supplementary Table 1). VA was significantly better in the XEN vs. the TRAB group after 12 months (*p* = 0.049), but did not differ at month 36 with mean VA values of 0.45 (0.25, 0.80) and 0.32 (0.21, 0.51) respectively (*P* = 0.387). Visual field did not differ at baseline nor any of the follow-up visits (all *P* > 0.05).

### Surgical success

Complete surgical success rates at month 12 through to month 36 and corresponding odds ratios comparing the TRAB group with the XEN group are shown in Supplementary Fig. 1. For instance, at month 24, complete success was achieved in 40.0% of patients receiving XEN vs. 62.8% of patients receiving TRAB, corresponding to an adjusted odds ratio of 2.70 (1.04–7.00, *p* = 0.040). At month 36, complete success was achieved in 27.3% after XEN and 56.8% after TRAB, yielding an adjusted odds ratio of 4.36 (1.25–15.18, *p* = 0.021). The findings for qualified surgical success, which expands the outcome definition to include cases requiring IOP-lowering medication in order to achieve target IOP values, are provided in the Supplementary Fig. 2.

### Postoperative interventions

**Fig. 3:**
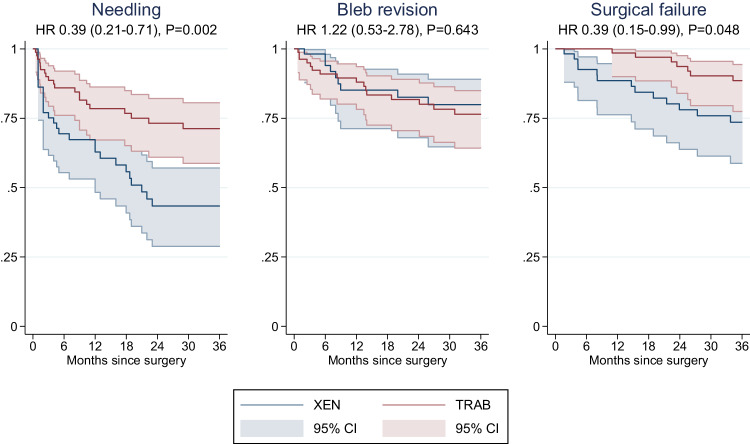
Kaplan-Meier curves showing the proportion free of needling, bleb revision, and surgical failure after XEN Gel Stent implantation or trabeculectomy. The hazard ratios presented in the subtitles were adjusted for age, sex, number of pre-operative IOP-lowering medications, and phacoemulsification. HR hazard ratio, TRAB trabeculectomy, XEN XEN Gel Stent.

The number of postoperative interventions is summarised in Table 2. Needling was less frequently performed in the TRAB group (20 eyes; 24%) than the XEN group (28 eyes; 48%) (*p* = 0.002). Four eyes in the TRAB group needed an anterior chamber (AC) filling with viscoelastic due to hypotony (2; 1.4%) or an AC rinsing due to a hyphaema (2; 1.4%), but no eye in the XEN group (*p* = 0.513). Only the TRAB group underwent suture lysis because of the trabeculectomy surgery’s intrinsic features that require sutures. There was no difference in the number of bleb revisions, bleb sutures due to hypotony, drainage of choroidal bleeding or additional transscleral cyclophotocoagulation. In time-to-event analyses (Fig. 3), the adjusted hazard ratio comparing the TRAB group with the XEN group was 0.39 for needling (95% CI 0.21–0.71, *p* = 0.002) and 1.22 for bleb revision (0.53–2.78, *p* = 0.643).

**Table 2:** Summary of postoperative interventions and repeat filtrating surgeries after surgical failure and ocular complications in the 36 months of follow-up.

	Total	XEN	TRAB	*P* value
	No. (column %)			
**Interventions**				
**Needling**				**0.002**
0x	94 (66.2)	30 (51.7)	64 (76.2)	
1x	33 (23.2)	18 (31.0)	15 (17.9)	
2x	13 (9.4)	10 (17.2)	3 (3.6)	
3x	2 (1.4)	0 (0.0)	2 (2.4)	
**Bleb revision**				1.00
0x	117 (82.4)	49 (84.75)	68 (80.1)	
1x	18 (12.7)	7 (12.0)	11 (13.1)	
2x	6 (4.2)	2 (3.5)	4 (4.8)	
3x	1 (0.7)	0 (0.00)	1 (1.2)	
**Bleb sutures due to hypotony**				1.00
0x	138 (97.2)	57 (98.3)	81 (96.4)	
1x	3 (2.1)	1 (1.7)	2 (2.4)	
2x	1 (0.7)	0 (0.0)	1 (1.2)	
**Laser suture lysis**				0.001
0x	127(89.4)	58 (100)	69 (82.1)	
1x	13 (9.2)	0 (0.0)	13 (9.12)	
2x	2 (1.4)	0 (0.0)	2 (1.4)	
**Filling AC with viscoelastics**				0.513
0x	140 (98.6)	58 (100)	82 (97.6)	
1x	2 (1.4)	0 (0.0)	2 (2.4)	
**AC rinsing**				0.513
0x	140 (98.6)	58 (100)	82 (97.6)	
1x	2 (1.4)	0 (0.0)	2 (2.4)	
**Drainage of choroidal effusion**				1.000
0x	141 (99.3)	58 (100)	82 (97.6)	
1x	1 (0.7)	0 (0.0)	2 (2.4)	
**TCP**				1.000
0x	139 (97.9)	57 (98.3)	82 (97.7)	
1x	3 (2.1)	1 (1.7)	2 (2.4)	
**Repeat filtrating surgeries**				
XEN	6 (4.2)	2 (3.5)	4 (4.8)	1.000
TRAB	10 (7.0)	9 (15.5)	1 (1.2)	**0.001**
Preserflo Microshunt implantation	4 (2.8)	2 (3.5)	2 (2.4)	1.000
Any type	20 (14.1)	13 (22.4)	7 (8.3)	0.026
**Complications**				
**No. of complications**				0.367
None	107 (75.4)	42 (72.4)	65 (77.4)	
One	29 (20.4)	15 (25.9)	14 (16.7)	
Two	5 (3.5)	1 (1.7)	4 (4.8)	
Four	1 (0.7)	0 (0.0)	1 (1.2)	
**Type of complication**				
Hypotony with choroidal effusion	19 (13.4)	8 (13.8)	11 (13.0)	0.882
Hyphaema	11 (7.8)	4 (6.9)	7 (8.3)	1.000
Corneal Erosion	2 (1.4)	0 (0.0)	2 (2.4)	0.513
Corneal Endothelial Dysfunction	1 (0.7)	1 (1.7)	0 (0.0)	0.408
XEN Dislocation	1 (0.7)	1 (1.7)	0 (0.0)	0.408
XEN Fracture	1 (0.7)	1 (1.7)	0 (0.0)	0.408
IOL-Capture	1 (0.7)	1 (1.7)	0 (0.0)	0.408
Glaucoma attack	1 (0.7)	1 (1.7)	0 (0.0)	0.408
Hypotonic maculopathy	1 (0.7)	0 (0.0)	1 (1.2)	1.000
Vessel Occlusion	1 (0.7)	0 (0.0)	1 (1.2)	1.000
Uveitis	1 (0.7)	0 (0.0)	1 (1.2)	1.000

*P* values were calculated using Fisher’s exact tests. Percentages may not sum up to 100.0% due to rounding.

*AC* anterior chamber, *IOL* intraocular lens, *TCP* transsceral cyclophotocoagulation, *TRAB* trabeculectomy, *XEN* XEN Gel Stent.

Bold values indicate statistical significance *p* < 0.05.

### Complete surgical failure

Complete surgical failure occurred in 20 out of 142 eyes (Table 2). Cumulative incidence of complete surgical failure was 22.4% in the XEN group (i.e. 13 of 58 eyes) and 8.3% in the TRAB group (i.e. 7 of 84 eyes). 10 failed cases received a trabeculectomy, 6 cases a XEN Gel Stent implantation and 4 cases a Preserflo Microshunt implantation as a secondary filtering glaucoma surgery, but there was no case of lost light perception. In the XEN group, 3 cases failed due to problems with the XEN Stent (2 XEN Dislocations, 1 XEN Fracture), in one case the XEN Gel Stent implantation was aborted due to a massive subtenon bleeding and a trabeculectomy was performed one month after the aborted surgery. All other cases failed due to bleb scaring. The Kaplan-Meier plot comparing incidence of surgical failure by treatment group is provided in Fig. 3. Compared to the XEN group, the adjusted hazard ratio in the TRAB group for suffering complete surgical failure was 0.39 (95% CI: 0.15–0.99, *P* = 0.048).

### Ocular complications

In 16 eyes (27.6%) in the XEN group and in 19 (22.7%) in the TRAB group an ocular complication was documented (Table 2), with no significant difference between the two procedures (*p* = 0.367). The most frequently reported postoperative complications were hypotony with choroidal effusion (XEN 13.8%, TRAB 13.0%, *p* = 0.882), and hyphaema (XEN 6.9%, TRAB 8.3%; *p* = 1.00) mostly self-resolving within 7 days postoperatively. One patient in the trabeculectomy group developed hypotony 27 months after initial surgery following a bleb revision. In 2 XEN patients (3.4%), stent problems were recorded. One stent migrated from the anterior chamber into the subconjunctival space, and one fractured within the first month after implantation. One XEN patient developed a glaucoma attack at month 6, with an IOP > 50 mmHg, one had to undergo Descement Membrane Endothelial Keratoplasty (DMEK) 15-months after XEN implantation due to a corneal endothelial dysfunction. In the TRAB group, one patient had choroidal effusion and retinal detachment after IOL fixation with the Yamane technique and had to undergo transscleral drainage of choroidal effusion and a pars plana vitrectomy. One TRAB patients developed a hypotonic maculopathy. One patient had a central retinal venous occlusion 7 month after surgery and one patient developed an anterior uveitis 6 month after surgery. One patient in each group had an IOL capture after phacoemulsification.

## Discussion

The results of this study suggest that both procedures significantly reduce intraocular pressure and the need of antihypertensive medications during a three year follow up period. Trabeculectomy is superior to the XEN Gel stent implantation in terms of intraocular pressure decrease as well as the 1-year necessity of postoperative medications, both with similar complication rates. In our study mean IOP in the XEN group decreased from 23.4 mmHg (95% CI 21.2–25.6) to 16.0 mmHg (13.2–18.7) 24 months and to 13.8 mmHg (12.0–5.6) 36 months postoperatively. In patients who received trabeculectomy mean IOP dropped from 25.1 mmHg (23.3–26.9) to 12.3 mmHg (10.6–14.0) at 24 month and 11.2 mmHg (9.6–12.8) at 36 months of follow up. The difference between XEN and TRAB becomes even more pronounced when age, sex, number of pre-operative IOP-lowering medications, pseudophakia and the fact that in some patients both eyes were included, were taken into account. The magnitudes of IOP decrease in both groups of our study are comparable with the retrospective cohort study by Wagner et al. (171 eyes, 82 XEN, 89 TRAB patients) that showed a reduction of IOP 7.2 ± 8.2 mmHg in the XEN group and 10.5 ± 9.2 mmHg in the trabeculectomy group 12 month postoperatively. However, consistent with our data the IOP decrease was significantly higher in the trabeculectomy group at 12 month follow up (*p* = 0.03) [8]. Similar to our study, Marco Parras et al. conducted a 36-month follow-up study comparing XEN Gel stent with trabeculectomy in patients with open-angle glaucoma (141 eyes XEN and 71 TRAB patients). In this study, IOP was significantly lower at every time point measured in the TRAB surgery group than in the XEN group (*p* < 0.001) [11]. Nevertheless, we achieved greater postoperative IOP reductions in both groups (XEN Gel Stent mean reduction 20% 12 months, 19% 24 months, 35% 36 months; TRAB group 50% 12 months, 48% 24 months, 50% 36 months). Our results are consistent with those published by Grover et al. (mean reduction 35.6% after 12 months) and Reitsamer et al. (mean reduction 29.3% after 12 months, 27,8% after 24 months) [16, 17]. Absolute mean postoperative IOP values of 11.0 and 15.5 mmHg 12 months after trabeculectomy in literature are comparable to our results [18, 19].

The number of antiglaucoma medications in our study decreased significantly in both groups. After 12 months the trabeculectomy group needed significantly less medication than the XEN group (2.0% vs. 43.2%, *p* < 0.001), but did not show a significant difference in the following two years of follow-up. 42.4% patients in the XEN Group vs. 68.6% in the TRAB group were free of IOP lowering medication at month 36 (*p* = 0.083). This result is suggestive of an inferiority of the XEN Gel stent in terms of the number of postoperative anti-glaucoma medications needed in the early postoperative phase. In line with our results, other studies have shown that fewer drops were taken after glaucoma surgery. Gillmann et al. showed a decrease in mean number of glaucoma medications from 2.0 ± 1.2 after XEN Gel in patients suffering from primary open angle glaucoma and from 2.0 ± 1.3 in pseudoexfoliation glaucoma patients at baseline to 0.6 ± 0.9 and 0.4 ± 0.7 at 24 months after XEN Gel Stent implantation, respectively [20]. Marcos Parra et al. showed a significant reduction of mean number of anti-glaucoma medication in the XEN and TRAB group (−1.8 and −2.1, respectively), but could not show a significant difference between the two groups after 36 months (*p* = 0.784). However, the evolution of the mean number of IOP-lowering medication showed a tendency to increase over time, more pronounced for the XEN45 alone group [11]. This supports our theory that the XEN Gel Stent is becoming less effective over time.

Our study showed a complete success rate of 27.3% in the XEN implant group and 56.8% in the TRAB group 36 months postoperatively. Marcos Parras et al. applied similar criteria for success (postoperative IOP reduction of ≥20% from preoperative baseline without any glaucoma medication). In their study eyes subjected to XEN were not inferior to that of trabeculectomy (49.2% vs. 66%, respectively, *p* = 0.0638) 12 months after XEN Gel Stent Implantation [11]. This differs from our data, resulting in a surgical success rate of 37.2% in the XEN group and 83.0% in the TRAB group 12 month postoperatively. The adjusted odds ratio of achieving surgical success after TRAB vs. XEN in our study is highest in the first year (OR = 12.64 [4.09. 39.06], *p* < 0.001). Even though it decreases over the 3-year observation period, it is still significantly increased after 36 months (OR = 4.36 [1.25, 15.18], *p* = 0.021). Consistent with our findings Cappelli et al. showed in a study analysing 68 eyes with open angle glaucoma (34 XEN, 34 TRAB) that trabeculectomy offers a higher probability of maintaining lower IOP values (≥6 to ≤12 mmHg) at 3 years than the XEN Gel Implant (*p* = 0.006) [13]. In the current study, the surgical failure rate, according to the criteria of Lenzhofer et al. [21], was significantly lower in eyes subjected to TRAB than to XEN (12.9% vs. 28.2%) with a hazard ratio for failure of 0.37 (0.15–0.89; *p* = 0.027). Lenzhofer et al. found a surgical failure rate of 10% per year within the first 5 years in the XEN Gel Stent group [21], consistent with the present findings. In the tube versus trabeculectomy study of Gedde et al., a failure rate of 28% after trabeculectomy has been observed at 3 years [22], which is considerable higher than compared to our results. However, the difference might be due to the fact, that relative or absolute IOP reduction criteria were excluded from the definition of surgical failure in our study [10, 21–23].

If necessary, needling or bleb revision to restore bleb function can be effective methods in the postoperative management of XEN Gel stent implantation or trabeculectomy. In the present study, eyes after XEN Gel Stent implantation received significantly more needling than TRAB patients (48% XEN vs. 24% TRAB, *p* = 0.001). The hazard ratio for first needling after TRAB versus XEN (HR = 0.39; 0.21–0.71; *p* = 0.002) shows that XEN patients received needling earlier and more frequently. The high needling rates after XEN Gel Stent implantation are consist with the results of Gillmann et al. who reported that in 55.4% eyes a needling had to be performed within 3 years after XEN implantation [20]. In other studies with a shorter follow-up time needling rates lay between 20% and 41.1% [7, 10, 21, 24]. The high rate of needling after XEN Gel Stent implantation compared to TRAB could be due to the fact that trabeculectomy produces a larger drainage area compared to the focal aqueous flow through a XEN Gel Stent [24]. It has also been postulated, that the tenon capsule itself may occlude the stent distally, especially if the XEN is not precisely placed in the subconjunctival space [23, 24]. In contrast, the opening of the conjunctiva in trabeculectomy not only facilitates the precise dissection of the subtenon space, but allows the tenon to be stretched and pulled forward with the limbal sutures that are set. Lenzhofer et al. demonstrated in a study assessing the outer stent position of the XEN Gel Stent in 66 eyes a higher efficacy and lower secondary needling rate in deeper implant positions (intra- and subtenon) in combination with primary needlings [25]. Therefore, the fact that we did not perform primary needling at the time of XEN implantations may have contributed to the high needling rate.

Overall, the rates of complications were low and similar in both groups. The most common complication in both groups was ocular hypotony followed by postoperative hyphaema. Most of the complications were self-limiting and resolved spontaneously. In the XEN group in two hypotonic eyes the AC was filled with viscoelastic to stabilise the eye and two hyphaema were rinsed. Our results did not significantly differ from those reported by Schlenker et al. [10]. In comparison, Marco Parras et al. and Sharpe et al. reported a higher incidence of hyphaema in the TRAB group compared to the XEN group [11, 24]. However, we cannot confirm these observations in our study. Our results suggest that in terms of postoperative complications trabeculectomy is not inferior to XEN, even though XEN is considered the less invasive procedure.

There are several limitations to this study, mainly related to its retrospective nature. A retrospective design always carries the risk of selection and information bias. However, involving subsequent patients should have minimised any selection bias. An ideal study to compare the two methods would be an RCT in which each eye is randomised to a different operation. As our study is a retrospective analysis, a limited number of patients had a TRAB in one eye and an XEN in the other eye and we were not able to perform an analysis or draw any conclusions. Heterogeneity resulting from the involvement of two different forms of glaucoma should be small, because it is well known, that the decrease in IOP is similar in patients with primary open angle and pseudoexfoliation glaucoma [9, 21]. In our study there was no evidence that the results differed between patients with primary open angle vs. pseudoexfoliation glaucoma. Furthermore, only a subset of participants had information on the primary endpoint assessed after 3 years of follow-up, but this is understandable against the background of a private practice ophthalmologist follow up-policy, who refer patients back to our clinic in case of untreatable high IOP or complications. As we are the only public hospital in our state performing glaucoma surgeries, it is unlikely that patients have been receiving glaucoma-related care in another hospital. Despite these reduction in sample size over the observational period, we were able to demonstrate significant results throughout the entire follow up of 36 months. Furthermore, we conducted a linear mixed model with repeated measurement taking this limitation and the fact that in a limited number of patients both eyes were included in the analysis into account, which further emphasised the difference between the two groups. The single centre setting of our study can be regarded as a strength, because all procedures were performed homogenously by only 2 highly experienced surgeons.

In conclusion, both XEN Gel Stent and trabeculectomy revealed to be safe and effective in lowering IOP and the number of IOP lowering medication needed. Trabeculectomy was more effective both in terms of IOP reduction, and reduction of the number of anti-glaucoma medications. The probability of achieving complete surgical success was four times higher in the TRAB group than in the XEN group 36 months postoperatively. Regular conjunctival assessments and bleb intervention are a necessity to guarantee long term success.

Supplemental material is available at Eye’s website.

## Summary

### What was known before

- Current evidence on the benefits and safety of XEN Gel Stent application compared to trabeculectomy in glaucoma patients is limited.

### What this study adds

- To address the uncertainty about the outcome of glaucoma patients over the longer term, we conducted a comprehensive retrospective study involving 142 eyes in 135 patients with repeated follow-up examination over a period of up to 36 months.
- This study shows that the reduction of intraocular pressure that was achieved by trabeculectomy was substantially and significantly higher than by XEN Gel Stent implantation.
- Furthermore, trabeculectomy was associated with less need for needling as well as a higher probability of achieving complete surgical success.

### Supplementary information

Supplementary Table 1: Intraocular pressure, within-person percentage reduction, visual acuity, and visual field and their differences between treatments groups over the 36-month follow-up.

Supplementary Figure 1: Unadjusted and multivariable adjusted* odds ratios for complete surgical success comparing trabeculectomy vs. XEN Gel Stent implantation. Adjusted for adjusted for age, sex, number of pre-operative IOP-lowering medications, and phacoemulsification. Abbreviations: CI=confidence interval; TRAB=trabeculectomy; XEN=XEN Gel Stent.

Supplementary Figure 2: Unadjusted and multivariable adjusted* odds ratios for qualified surgical success comparing trabeculectomy vs. XEN Gel Stent implantation. *Adjusted for adjusted for age, sex, number of pre-operative IOP-lowering medications, and phacoemulsification. Abbreviations: CI=confidence interval; TRAB=trabeculectomy; XEN=XEN Gel Stent.

## Data Availability

The data of this study are available on request from the corresponding author.
