# Supplementary material for: Three-year clinical outcome of XEN45 Gel Stent implantation versus trabeculectomy in patients with open angle glaucoma
Source: Eye (Lond). 2024 Mar 28;38(10):1908–16. doi: 10.1038/s41433-024-03042-z (PMC11226636; doi:10.1038/s41433-024-03042-z)
Supplement: Supplementary file 1 — Supplementary Table 1: Intraocular pressure, within-person percentage reduction, visual acuity, and visual field and their differences between treatments groups over the 36-month follow-up. [file 41433_2024_3042_MOESM1_ESM.docx]

Supplementary Table 1 – **Intraocular pressure, within-person percentage reduction, visual acuity, and visual field and their differences between treatments groups over the 36-month follow-up.**

| **Time point** | **XEN** | |  | **TRAB** | |  | **TRAB vs. XEN** | |
| --- | --- | --- | --- | --- | --- | --- | --- | --- |
|  |  | Mean (95% CI) |  | n | Mean (95% CI) |  | Mean difference (95% CI) | P value |
| **PreOP** |  |  |  |  |  |  |  |  |
| IOP (mmHg) | 58 | 23.4 (21.2, 25.6) |  | 84 | 25.1 (23.3, 26.9) |  | 1.7 (-1.1, 4.5) | 0.245 |
| Mean defect | 55 | 14.4 (12.4, 16.5) |  | 79 | 16.5 (15.1, 17.9) |  | 2.1 (-0.3, 4.5) | 0.093 |
| Visual acuity* | 58 | 0.38 (0.29, 0.50) |  | 84 | 0.34 (0.28, 0.41) |  | -10% (-35, 24) | 0.518 |
| **Day 1-3** |  |  |  |  |  |  |  |  |
| IOP (mmHg) | 51 | 7.1 (6.1, 8.0) |  | 81 | 11.4 (10.0, 12.8) |  | 4.3 (2.4, 6.2) | **<0.001** |
| % reduction vs. preOP | 51 | 66% (60, 73) |  | 81 | 50% (43, 57) |  | -16% (-26, -6) | **0.002** |
| Visual acuity* | 35 | 0.28 (0.21, 0.38) |  | 71 | 0.13 (0.10, 0.17) |  | -54% (-70, -30) | **0.001** |
| **Week 1** |  |  |  |  |  |  |  |  |
| IOP (mmHg) | 42 | 10.3 (9.0, 11.6) |  | 47 | 11.7 (10.0, 13.3) |  | 1.4 (-0.8, 3.5) | 0.214 |
| % reduction vs. preOP | 42 | 51% (43, 58) |  | 47 | 49% (41, 58) |  | -2% (-13, 10) | 0.798 |
| Visual acuity* | 24 | 0.42 (0.32, 0.56) |  | 29 | 0.26 (0.20, 0.36) |  | -37% (-59, -5) | **0.031** |
| **Month 1** |  |  |  |  |  |  |  |  |
| IOP (mmHg) | 56 | 16.6 (14.7, 18.6) |  | 55 | 10.6 (8.8, 12.3) |  | -6.1 (-8.7, -3.4) | **<0.001** |
| % reduction vs. preOP | 56 | 21% (9, 32) |  | 55 | 53% (44, 62) |  | 32% (17, 47) | **<0.001** |
| Visual acuity* | 39 | 0.42 (0.31, 0.56) |  | 44 | 0.29 (0.22, 0.39) |  | -31% (-54, 5) | 0.084 |
| **Month 3** |  |  |  |  |  |  |  |  |
| IOP (mmHg) | 54 | 16.5 (14.9, 18.1) |  | 58 | 10.5 (9.1, 11.8) |  | -6.1 (-8.2, -4.0) | **<0.001** |
| % reduction vs. preOP | 54 | 19% (7, 32) |  | 58 | 55% (48, 61) |  | 35% (22, 49) | **<0.001** |
| Visual acuity* | 41 | 0.52 (0.40, 0.68) |  | 46 | 0.32 (0.23, 0.44) |  | -39% (-60, -6) | **0.028** |
| **Month 6** |  |  |  |  |  |  |  |  |
| IOP (mmHg) | 52 | 16.6 (14.2, 19.0) |  | 59 | 12.2 (10.2, 14.2) |  | -4.4 (-7.4, -1.3) | **0.006** |
| % reduction vs. preOP | 52 | 24% (11, 36) |  | 59 | 47% (37, 57) |  | 23% (7, 39) | **0.005** |
| Mean defect | 18 | 11.8 (8.1, 15.4) |  | 17 | 14.3 (10.7, 17.9) |  | 2.6 (-2.6, 7.7) | 0.335 |
| Visual acuity* | 40 | 0.49 (0.37, 0.66) |  | 47 | 0.40 (0.32, 0.50) |  | -18% (-43, 17) | 0.269 |
| **Month 12** |  |  |  |  |  |  |  |  |
| IOP (mmHg) | 43 | 15.8 (13.9, 17.7) |  | 54 | 10.9 (9.5, 12.2) |  | -5.0 (-7.2, -2.7) | **<0.001** |
| % reduction vs. preOP | 43 | 20% (1, 39) |  | 54 | 50% (42, 58) |  | 30% (11, 49) | **0.003** |
| Mean defect | 21 | 12.3 (9.4, 15.3) |  | 20 | 15.4 (12.1, 18.8) |  | 3.1 (-1.4, 7.5) | 0.184 |
| Visual acuity* | 36 | 0.54 (0.44, 0.66) |  | 48 | 0.38 (0.30, 0.49) |  | -29% (-49, -1) | **0.049** |
| **Month 18** |  |  |  |  |  |  |  |  |
| IOP (mmHg) | 38 | 13.6 (12.6, 14.6) |  | 43 | 11.2 (9.4, 12.9) |  | -2.5 (-4.5, -0.4) | **0.022** |
| % reduction vs. preOP | 38 | 38% (30, 45) |  | 43 | 51% (41, 60) |  | 13% (1, 26) | **0.037** |
| Mean defect | 12 | 13.7 (10.2, 17.2) |  | 9 | 14.3 (10.4, 18.3) |  | 0.7 (-4.6, 6.0) | 0.804 |
| Visual acuity* | 28 | 0.43 (0.30, 0.62) |  | 38 | 0.33 (0.23, 0.47) |  | -24% (-55, 28) | **0.306** |
| **Month 24** |  |  |  |  |  |  |  |  |
| IOP (mmHg) | 35 | 16.0 (13.2, 18.7) |  | 44 | 12.3 (10.6, 14.0) |  | -3.7 (-6.8, -0.6) | **0.023** |
| % reduction vs. preOP | 35 | 19% (-4, 42) |  | 44 | 48% (38, 58) |  | 29% (6, 52) | **0.018** |
| Mean defect | 15 | 11.8 (8.2, 15.5) |  | 14 | 13.7 (9.4, 18.0) |  | 1.9 (-3.8, 7.5) | 0.520 |
| Visual acuity* | 24 | 0.59 (0.46, 0.75) |  | 35 | 0.33 (0.22, 0.52) |  | -43% (-67, -0) | 0.055 |
| **Month 30** |  |  |  |  |  |  |  |  |
| IOP (mmHg) | 21 | 14.0 (11.8, 16.3) |  | 38 | 12.1 (10.3, 13.8) |  | -2.0 (-4.8, 0.9) | 0.182 |
| % reduction vs. preOP | 21 | 37% (25, 49) |  | 38 | 50% (42, 58) |  | 13% (-1, 27) | **0.073** |
| Mean defect | 8 | 13.2 (6.8, 19.5) |  | 12 | 16.9 (14.2, 19.7) |  | 3.7 (-2.4, 9.9) | 0.249 |
| Visual acuity* | 15 | 0.62 (0.45, 0.86) |  | 28 | 0.42 (0.29, 0.62) |  | -32% (-62, 22) | 0.202 |
| **Month 36** |  |  |  |  |  |  |  |  |
| IOP (mmHg) | 22 | 13.8 (12.0, 15.6) |  | 38 | 11.2 (9.6, 12.8) |  | -2.6 (-5.1, -0.1) | **0.048** |
| % reduction vs. preOP | 22 | 35% (23, 48) |  | 38 | 50% (41, 60) |  | 15% (0, 31) | 0.054 |
| Mean defect | 11 | 13.4 (8.8, 18.0) |  | 16 | 14.4 (11.3, 17.4) |  | 1.0 (-4.3, 6.3) | 0.724 |
| Visual acuity* | 17 | 0.45 (0.25, 0.80) |  | 30 | 0.32 (0.21, 0.51) |  | -28% (-66, 51) | 0.387 |

*Visual acuity was summarised as geometric mean and 95% confidence interval because of its skewed distribution. Abbreviations: CI=confidence interval; IOP=intraocular pressure.
