# Supplementary figures and images for: Three-year clinical outcome of XEN45 Gel Stent implantation versus trabeculectomy in patients with open angle glaucoma
Source: Eye (Lond). 2024 Mar 28;38(10):1908–16. doi: 10.1038/s41433-024-03042-z (PMC11226636; doi:10.1038/s41433-024-03042-z)

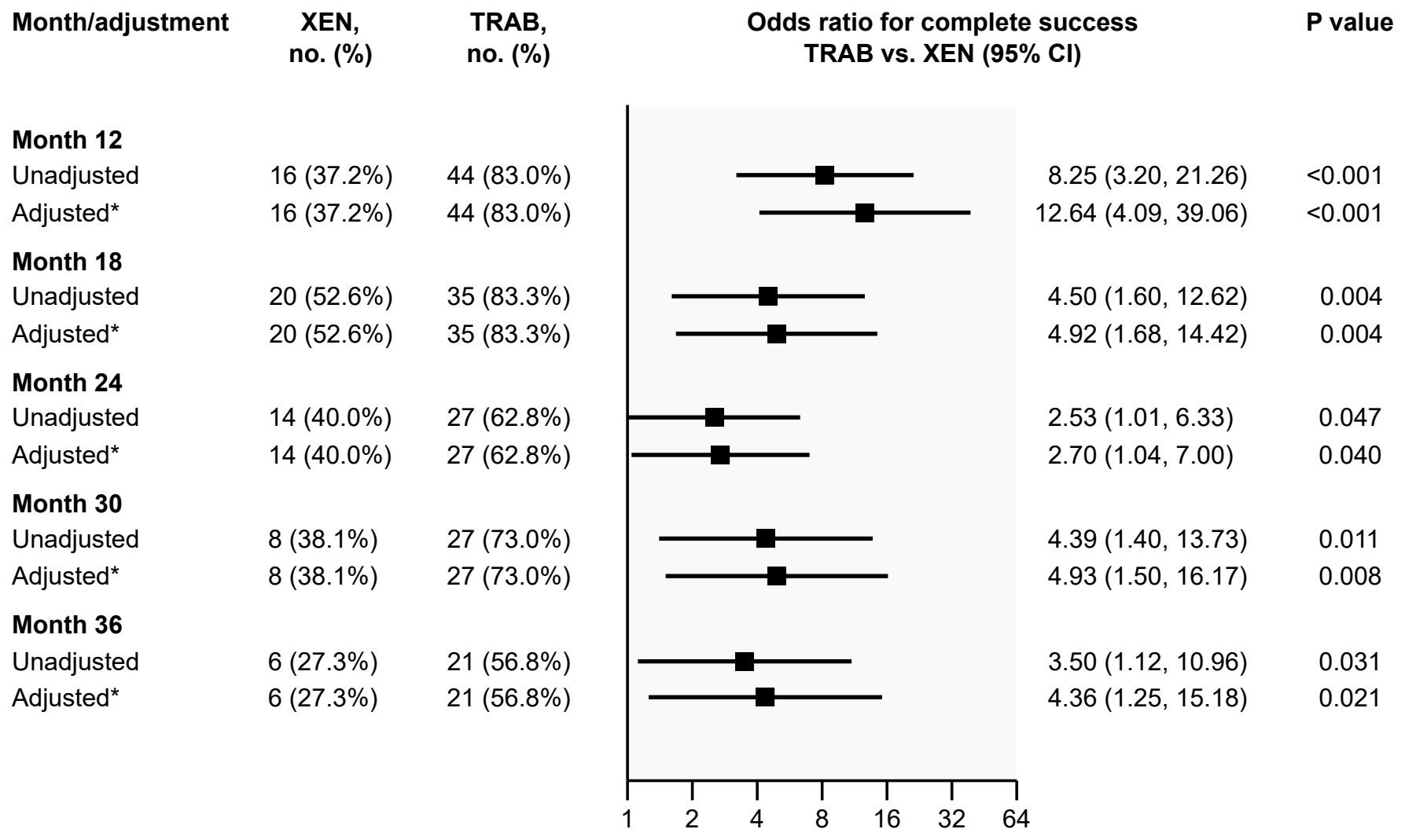

Supplement: Supplementary file 2 — Supplementary Figure 1: Unadjusted and multivariable adjusted* odds ratios for complete surgical success comparing trabeculectomy vs. XEN Gel Stent implantation. Adjusted for adjusted for age, sex, number of pre-operative IOP-lowering medications, and phacoemulsification. Abbreviations: CI=confidence interval; TRAB=trabeculectomy; XEN=XEN Gel Stent. [file 41433_2024_3042_MOESM2_ESM.pdf]

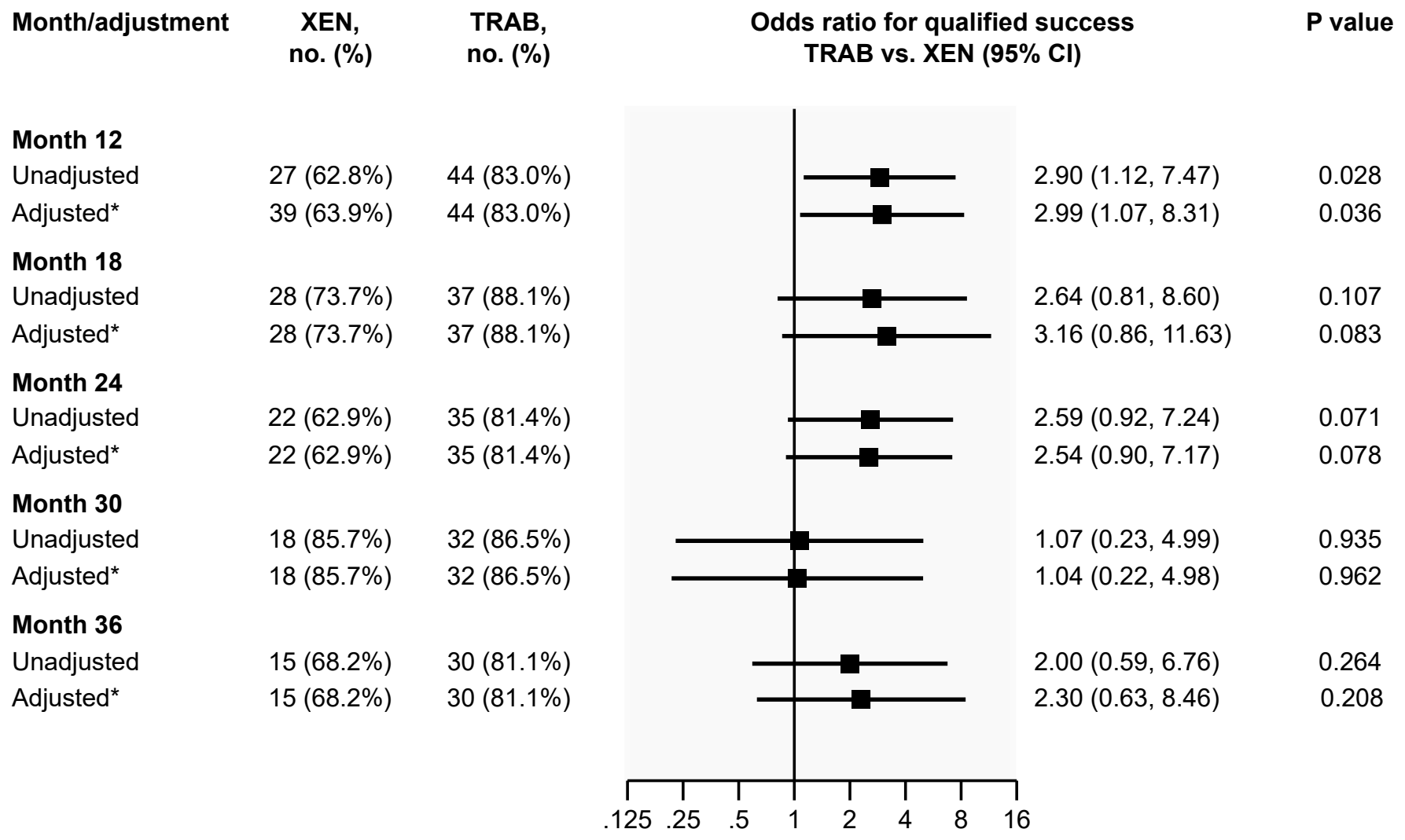

Supplement: Supplementary file 3 — Supplementary Figure 2: Unadjusted and multivariable adjusted* odds ratios for qualified surgical success comparing trabeculectomy vs. XEN Gel Stent implantation. *Adjusted for adjusted for age, sex, number of pre-operative IOP-lowering medications, and phacoemulsification. Abbreviations: CI=confidence interval; TRAB=trabeculectomy; XEN=XEN Gel Stent. [file 41433_2024_3042_MOESM3_ESM.pdf]
